# Nonlinear analysis of EEG complexity in episode and remission phase of recurrent depression

**DOI:** 10.1002/mpr.1816

**Published:** 2019-12-09

**Authors:** Milena Čukić, Miodrag Stokić, Slavoljub Radenković, Miloš Ljubisavljević, Slobodan Simić, Danka Savić

**Affiliations:** ^1^ Department of General Physiology and Biophysics, School of Biology University of Belgrade Belgrade Serbia; ^2^ Cognitive Neuroscience Department, Life Activities Advancement Center Belgrade Serbia; ^3^ Division for Back‐End Development, TomTom Amsterdam Netherlands; ^4^ Department of Physiology, College of Medicine and Health Sciences UAE University Al Ain United Arab Emirates; ^5^ Department for Forensic Psychiatry, Institute for Mental Health Belgrade Serbia; ^6^ Laboratory of Theoretical and Condensed Matter Physics 020/2, Vinča Institute University of Belgrade Belgrade Serbia

**Keywords:** complexity, electroencephalogram, Higuchi's fractal dimension, recurrent depression, sample entropy

## Abstract

**Objectives:**

Biomarkers of major depressive disorder (MDD), its phases and forms have long been sought. Objectives were to examine whether the complexity of EEG activity, measured by Higuchi's fractal dimension (HFD) and sample entropy (SampEn), differs between healthy subjects, patients in remission, and in episode phase of the recurrent depression and whether the changes are differentially distributed between hemispheres and cortical regions.

**Methods:**

Resting state EEG with eyes closed was recorded from 22 patients suffering from recurrent depression (11 in remission, 11 in the episode), and 20 age and sex‐matched healthy control subjects. Artifact‐free EEG epochs were analyzed by in‐house developed programs running HFD and SampEn algorithms.

**Results:**

Depressed patients had higher HFD and SampEn complexity compared to healthy subjects. The complexity was higher in patients who were in remission than in those in the acute episode. Altered complexity was present in the frontal and centro‐parietal regions when compared to control group. The complexity in frontal and parietal regions differed between the two phases of depressive disorder.

**Conclusions:**

Complexity measures of EEG distinguish between the healthy controls, patients in remission and episode. Further studies are needed to establish whether these measures carry a potential to aid clinically relevant decisions about depression.

## 1 INTRODUCTION

Major depressive disorder (MDD) is a serious mental ill health associated with protracted personal suffering and significant social and functional impairment. It has become the leading cause of ill health and disability worldwide (World Health Organization, 2017). Depression has a strong tendency to reoccur—a significant number of patients will suffer from at least one more episode after the first one, reaching four episodes on average during the lifetime (Solomon et al., 2000). In these patients, the risk of new episodes rises significantly with each subsequent recurrence although the course of the disease can be unique (Solomon et al., 2000). Hence, the decision to stop the therapy, or to initiate the maintenance therapy to prevent a relapse in patients with recurrent depression who have achieved remission, often presents a significant clinical challenge (Teasdale et al., 2000). Therefore, finding accurate and reliable biomarkers that can help differentiate MDD episode from remission is of considerable importance.

Contrary to other specializations in medicine, psychiatry at the moment does not use objective diagnostic tests (Gillan & Daw, 2016; Shorter & Fink, 2010). Recently authors showed that relying on self‐report from the patient and personal experience of a clinician as the sole basis for diagnosis did not show to be so accurate (Gillan & Whelan, 2017; Rush et al., 2011). Also, psychiatry research online showed that automatization of collecting the self‐report from patients anytime anywhere showed that the role of the educated interviewer is not inevitable in the process (Berinsky, Huber, & Lenz, 2012). Computational psychiatry is showing many fruits already as a novel avenue of research. Our, as well as studies of other researchers, showed that automated detection of depression is possible and in the last decade many studies showed it to be the accurate and feasible methodology (Acharya et al., 2015; Ahmadlou, Adeli, & Adeli, 2012; Bachmann, Lass, Suhhova, & Hinrikus, 2013; Bachmann et al., 2018; Čukić et al., 2018; Faust, Chuan, Ang, Puthankattil, & Joseph, 2014; Hosseinifard, Moradi, & Rostami, 2014; Puthankattil & Joseph 2012). There is a need for data‐driven changes to the system of psychiatric diagnostics.

Another separate avenue of research, namely physiological complexity (based on Complex Dynamical Systems Theory) is also considered to be novel, but there is already sufficient evidence in the present literature that in depression, elevated complexity of EEG can be measured (Ahmadlou et al., 2012; Bachmann et al., 2013, 2018; Čukić et al., 2018; Čukić, Oommen, Mutavdzic, Jorgovanovic, & Ljubisavljevic, 2013; Lebiecka et al., 2018). Earlier research in MRI, fMRI, high‐density EEG graph‐theory application, and fractional anisotropy showed that in MDD a decreased functional connectivity can be observed (de Kwaasteniet et al., 2013; Kim, Bolbecker, & Howell, 2013; Vederine, Wessa, Leboyer, & Houenou, 2011) with a yet unknown cause. The possible consequence of deep white matter tracts deficit (the second part of uncinate fasciculus according to de Kwaasteniet et al. (2013)) may contribute to abnormal functional connectivity within fronto‐lymbic network. We can hypothesize that this confirmed structural change might lead to elevated excitability on the cortex. Several authors clearly stated in their work that this kind of increased physiological complexity can be measured in all the positions on standard EEG cap (Bachmann et al., 2013; Čukić et al., 2018; Hosseinifard et al., 2014). This is also in line with the research utilizing only classical spectral analysis (Fingelkruis et al., 2014; Fingelkurts et al., 2006; Fingelkurts & Fingelkurts, 2015). DeBattista et al. (2011) showed that EEG markers outperform clinical‐determined treatment plans for depression. There is almost a kind of the consensus among researchers that any of the fractal and nonlinear methods we use, can confirm an elevated complexity in patients diagnosed with depression.

One of the first studies about changes in complexity in depression (Nandrino et al., 1994) found that EEG in MDD can be more predictive using a correlation dimension (attractor reconstruction) analysis. However, they could not detect the changes between patients in the episode and remission. Ahmadlou et al. (2012) compared the detection performance of two different algorithms for the fractal dimension, showing Higuchi to be better when compared to Katz's algorithm. It needs to be mentioned that they used just prefrontal electrodes. Hosseinifard et al. (2014), among other nonlinear measures as features for further machine learning, used Higuchi fractal dimension showing elevated complexity as well. Bachmann et al. (2013) also detected elevated HFD in depression (the only exclusively female sample) mentioning that they confirmed it on all electrode positions, but opted to use just four electrodes for the sake of detection. The only study we are aware of to probe nonlinear measure (Lempel‐Ziv complexity) and stated not to find the differences with healthy controls was a study by Arns, Cerquera, Gutiérrez, Hasselman, and Freund (2014). Bachmann et al. (2018) applied the same algorithm finding significant results on a similar sample. The difference between these two studies was in choosing broadband signal instead of standard sub‐bands of EEG in the study of Bachmann et al. Puthankattil and Joseph (2012) as well as Akar, Kara, Agambayev, and Bilgic (2015) applied wavelet‐chaos methodology to explore complexity in both parietal and frontal regions in MDD patient's EEG. Both studies found an increased complexity in parietal and frontal regions in MDD patients' EEG compared to healthy control. Faust et al. (2014) compared several entropy measures for the same detection of complexity changes showing it to be highly useful for accurate machine learning. In our recent study, we applied HFD and sample entropy as features for depression detection task (Čukić et al., 2018) showing that high accuracy is possible. Previously we compared those two measures in methodological senses to compare their difference concerning frequent content of the signal (Čukić et al., 2018). Higuchi's fractal dimension (HFD) measures complexity directly in the time‐domain, and sample entropy (SampEn) is irregularity statistic, measuring the predictability of the signal under study. Consequently, we can interpret both measures in terms of complexity. In Complex Dynamical Systems Theory there are vast families of measures we could apply for this analysis, due to their different mathematical origin, but we opted to use this specific pair due to their applicability in real time. All electrophysiological signals are representing underlying processes that are typically nonlinear, nonstationary, and nonequilibrium in nature. Conventional analyses like applying means, standard deviations, and classical power spectrum analysis are simply not capable to detect the subtle changes in such a complex signal. Hence, nonlinear analysis is a better method for that. de la Torre‐Luque and Bornas (2017) in their review about the detection of complexity changes in depression concluded that “EEG dynamics for depressive patients appear more complex but might be more random than the dynamics of healthy nondepressed individuals.”

Complexity analysis is still considered “novel” because the classical spectral analysis is deeply rooted in electrophysiology and nonlinear analysis is not (Klonowski, 2007). Contrary to our previous research where we used nonlinear measures in combination with machine learning methodology. With present work, we aimed to show that the utilization of two simple nonlinear measures might be sufficient. This methodological couple of two nonlinear measures can yield additional test to clinicians to make better decisions on further therapeutic steps informed about the present physiological status of the patient. We analyzed anonymized data which is also one of General Data Protection Regulation (GDPR) requirements for a possible later web‐based application.

To the best of our knowledge, there is no similar publication regarding this particular differentiation between episode and remission phase in depression. The precise question of this research is not whether we can detect MDD from EEG (because it has been repeatedly shown to be possible) but to explore the possibility to differentiate between two phases of the disease based on nonlinear biomarkers extracted from EEG.

## 2 METHODS

### 2.1 Participants

EEG data were recorded at the Institute for Mental Health in Belgrade, Serbia. The initial sample comprised 26 patients (17 females and nine male) suffering from recurrent depression, 25–68 years old (mean 32.40, SD 10.16). However, the final sample comprised 22 patients due to excessive artefacts in EEG recordings of four patients. As a control, we used EEG recordings of 20 age‐matched (mean 30.14, SD 8.94) healthy controls (10 males, 10 females) with no history of any neurological or psychiatric disorders, recorded at the Institute for Experimental Phonetic and Speech Pathology in Belgrade, Serbia. All participants were right‐handed, according to Edinburgh Handedness Inventory. The participants were informed about the experimental protocol and signed informed consent. The protocol was approved by the Ethical Committees of the participating institutions. All participants with depression were on medications and under the supervision of an experienced specialist in clinical psychiatry. Their diagnoses were made according to ICD‐10 scale. The study compared three groups: healthy controls (C), and depressed patients (D) in an episode (E), and in remission (R). Half of the patients were recorded while they were in an acute episode, while the other half were in remission phase of the disease. Participants' data are given in Table 1.

**Table 1:** Participants' data

	C	E	R
*N*	20	11	11
Sex			
Male	10	4	5
Female	10	7	6
Age	30.14 (8.94)	40.1 (10.94)	44.64 (10.87)
Education			
Low (primary)	0	0	0
Medium (high school)	6 (30%)	4 (36.36%)	5 (45.45%)
High (BS, MS, PhD)	14 (70%)	7 (63.64%)	6 (54.54%)
ICD‐10 (MDI) score	–	36.27 (5.41)	31.73 (3.80)

*Note*: C, control group; E, patients in an acute episode; R, patients in remission phase, age is given in years (standard deviation); MDI, Major Depression Inventory (ICD‐10), the mean (standard deviation) is present.

### 2.2 Data acquisition

EEG was recorded in the resting state with 10/20 International system for electrode placement, using NicoletOne Digital EEG Amplifier (VIAYSYS Healthcare Inc. NeuroCare Group), with closed eyes without any stimulus (resting state EEG). EEGs were recorded from 19 electrodes in a monopolar montage (Electro‐cap International Inc., Eaton, OH). The sampling rate was 1 kHz. The resistance was kept less than 5 kΩ. A bandpass filter was 0.5–70 Hz. The same setup was used for the control group, using Nihon Kohden Inc. apparatus. Since the protocol of recordings was the same, the fact that we used recordings from two different EEG producers' apparata did not introduce the difference between the groups (Pivik et al., 1993).

Participants were instructed to reduce any movement, staying in a comfortable sitting position with eyes closed. Each recording session lasted for 3 min (180 s—180,000 samples). The EEG records of four subjects were discarded from further analyses because of the high level of muscle activity or blinking artifacts. Further, we used records from 22 patients and 20 healthy controls for this study.

### 2.3 EEG data preprocessing

From each recording a 5‐s segment for the analyses was selected from the beginning (25,000–30,000 samples), middle (85,000–90,000 samples), and the end of the recording (145,000–150,000 samples). Each epoch comprises of 5,000 samples. For preprocessing of the obtained EEG data, we have used infomax independent component analysis (ICA) with the use of EEGLAB (Delorme & Makeig, 2004) working under MATLAB. Those components that correlated with eye‐movement and eye‐blink artifacts were removed.

Therefore, for each subject, epoch, and electrode, two nonlinear measures were calculated.

### 2.4 Data analysis

Initially, the classical spectral analysis was performed by constructing spectral power maps (EEGLAB program; Delorme & Makeig, 2004). Further HFD and SampEn analyses were performed by algorithms written in Java programming language. PCA was performed in MATLAB. Thereon, for all EEG epochs, HFD and SampEn were calculated. Fractal and SampEn maps were constructed on the whole spectrum, not dividing the signal into bands. The analysis was adopted as it has been shown that the Fourier analysis is redundant to fractal analyses (Kalauzi, Bojić, & Vuckovic, 2012). The fractal dimension of EEG was calculated by using Higuchi's algorithm (Higuchi, 1988), demonstrated to be the most appropriate for electrophysiological data (Esteller, Vachtsevanos, Echauz, & Litt, 2001). This method provides a reasonable estimate of the fractal dimension even if short signal segments are analyzed and it is computationally fast. HFD was also chosen because it is widespread in the EEG literature facilitating comparison of the results. We performed the Higuchi's algorithm (Higuchi, 1988), with the maximal length of an epoch *k*_max_ = 8, shown to perform the best for this type of signals (Spasic, Kalauzi, Grbic, Martac, & Culic, 2005). HFD of a time series is a measure of its complexity and self‐similarity in the time domain. HFD is not an integer, and the value of fractal dimension (FD) of waveforms (e.g., EEG) can range between 1 and 2. Higher self‐similarity and complexity results in higher HFD (Eke, Herman, Kocsis, & Kozak, 2002). SampEn was computed according to the procedure by Richman and Moorman (2000). SampEn estimates the signal complexity by computing the conditional probability that two sequences of a given length *n*, similar for *m* points, remain similar within tolerance *r* at the next data point (when self‐matches are not included). SampEn measures the irregularity of the data (the higher the values, the less regular signal) that is related to signal complexity (Pincus, 2006). SampEn was calculated using tolerance level of *r* = 0.15 times the standard deviation of the time series and *m* = 2, shown to be optimal for EEG (Molina‐Picó et al., 2011). Both HFD and SampEn were calculated for each electrode for the duration of signal (the epochs of artifact‐free recorded EEG; three epochs from each recording), using the in‐house written algorithm in Java programming language. It should also be noted that correlations with any medical data were not explored since the main aim of the study was to find independent nonlinear markers based on analysis of the EEG signal, which could be utilized as an additional tool in clinical practice.

### 2.5 Statistical analyses

Both HFD and SampEn values were used as an ensemble for analysis of variance (ANOVA with post hoc Bonferroni correction, SPSS Statistics version 20.0, SPSS Inc.). The Kolmogorov Smirnov test showed that HFD and SampEn data were not normally distributed. To obtain data with normal distribution and to include it in the analysis of the difference in complexity in resting state EEG data, normalized values of SampEn and HFD obtained from epochs of recorded EEG were calculated as log10 normalization in SPSS. Normalized SampEn and HFD data were compared using ANOVA with factors state (Controls vs. Depression, and Controls vs. Episode vs. Remissions) and position of the electrode (1–19). For every electrode, ANOVA was repeated for each measure independently. Bonferroni correction was used where appropriate. For all analyses, probability values *p* = 0.05 were considered as statistically significant.

### 2.6 Principal component analysis (PCA)

To reduce the dimensionality of the problem and decorrelate the measures (HFD and SampEn calculated from the same epochs extracted from the raw EEG signal), we utilized PCA (Jolliffe, 1986) in order to obtain three principal components (PCs) corresponding to largest eigenvalues of the sample covariance matrix. We defined percentage of the explained variance by first three PCs as ratio between sums of variances of three PCs and original variables. Here we wanted to demonstrate the possibility of classification of previously calculated nonlinear measures, by utilizing only the first three components in order to see whether the data were separable. We used Matlab 15b for this calculation (MathWorks, MA).

## 3 RESULTS

### 3.1 Spectral power maps

**Figure 1:**
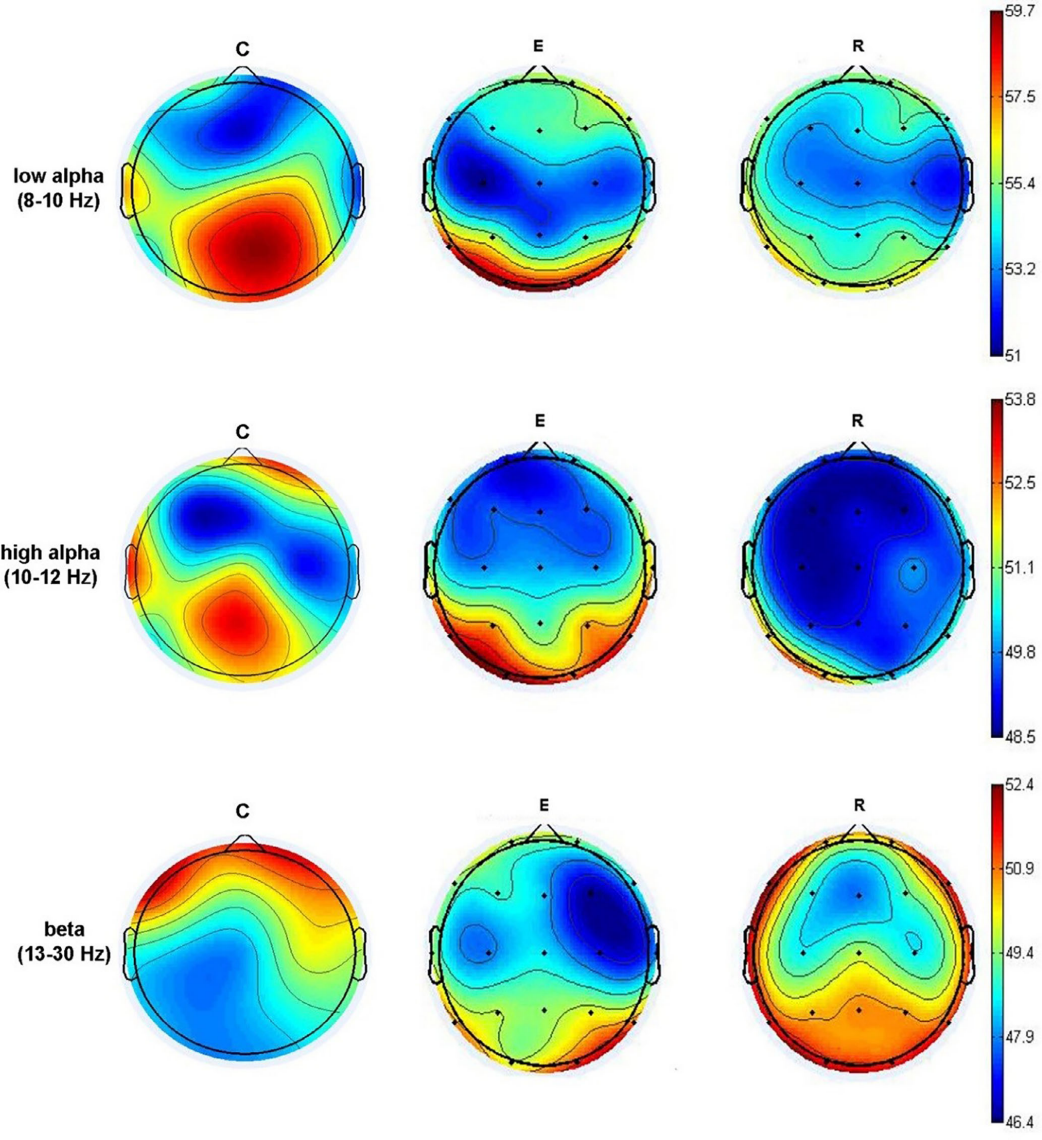
Spectral power maps are showing the difference between healthy persons (C) and those in the acute episode (E) and remission (R)

The first level of analysis was to compare spectral power maps of low alpha (8–10 Hz), high alpha (10–12 Hz), and beta (13‐30 Hz) bands between healthy controls (C) and patients in a different phase of the disease (i.e., episode—E or remission—R) (Figure 1).

Spectral power maps in low alpha band showed an overall decrease in both E and R groups in posterior regions (maximum at C3, C4, Cz, P3, P4, Pz, and T3) when compared to C group. In E and R groups, there was an increase in low alpha spectral power in the right prefrontal region (Fp2) and lateral right frontal region (F8) when compared to C group. Spectral power maps in high alpha band showed a decrease of high alpha spectral power in right prefrontal (Fp2), left temporal (T3), and central (C3, Cz) regions in E and R groups when compared to C group. Spectral power maps in beta band showed a decrease of beta (13–30 Hz) spectral power at frontal (Fz, Fp1, Fp2, F3, F4, F7, F8), and central‐temporal (C3, T3) regions in E and R groups when compared to C group. In contrast, there was an increase of beta spectral power in posterior regions (P3, P4, Pz, T5, T6, O1, O2) in E and R groups when compared to C group. In the E group only there was a frontal (F3 > F4) and temporal‐occipital (T5/O1 < T6/O2) asymmetry.

The results of ANOVA test for analysis of the group effect on the spectral power for each electrode location and frequency band are given in Table 2.

**Table 2:** Results of ANOVA test probing the effect of group on spectral power within low alpha, high alpha, and beta band for each electrode location

Electrode location	Low alpha (8–10 Hz)		High alpha (10–12 Hz)		Beta (13–30 Hz)	
	*F*(2,40)	*p*	*F*(2,40)	*p*	*F*(2,40)	*p*
*Fp1*	*	*	*	*	7.207	0.02
*Fp2*	*	*	9.211	0.01	8.107	0.01
*F3*	*	*	*	*	12.342	0.01
*F4*	*	*	*	*	18.674	0.01
*F7*	*	*	*	*	4.922	0.04
*F8*	*	*	*	*	6.817	0.03
*Fz*	*	*	*	*	7.997	0.02
*T3*	*	*	8.633	0.01	*	*
*T4*	*	*	*	*	*	*
*T5*	*	*	*	*	13.207	0.01
*T6*	*	*	*	*	14.338	0.01
*C3*	8.748	0.02	4.096	0.05	*	*
*C4*	5.168	0.04	*	*	*	*
*Cz*	6.381	0.03	10.734	0.01	*	*
*P3*	5.748	0.02	*	*	7.002	0.02
*P4*	4.286	0.05	*	*	6.699	0.03
*Pz*	*	*	*	*	14.361	0.01
*O1*	*	*	*	*	16.189	0.01
*O2*	*	*	*	*	16.917	0.01

* not significant difference.

### 3.2 HFD and sSampEn

**Figure 2:**
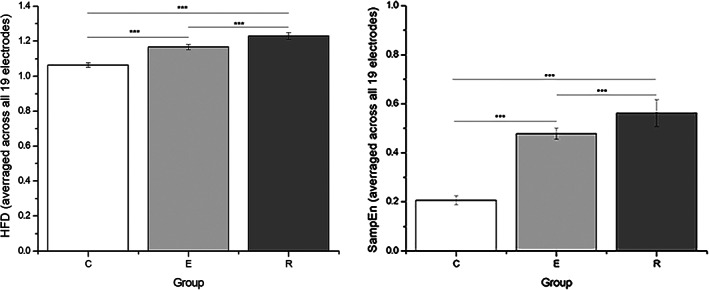
Values of HFD (left) and SampEn (right) averaged across all 19 electrodes, for all three groups (C: control; E: episode; and R: remission). Note the difference in scale for HFD and SampEn. ***p < 0.01. HFD, Higuchi's fractal dimension

Figure 2 summarizes the difference in HFD and SampEn values in all three groups averaged across all electrodes. HFD values for healthy controls ranged from 1.0334 to 1.15 (mean 1.064), for patients in the episode from 1.0586 to 1.3978 (mean 1.1664), and for those in remission from 1.0579 to 1.4938 (mean 1.2299). SampEn values for healthy controls were 0.14–0.32 (mean 0.2066), in the episode group 0.27–0.71 (mean 0.4783) and in remission group 0.34–0.74 (mean 0.5626).

ANOVA showed a significant effect of group on HFD (*F*[2,123] = 53.545, *p* < 0.001) and on SampEn values (*F*[2,123] = 190.864, *p* < 0.001). The post hoc test showed that both HFD and SampEn values were lowest in the C group, followed by participants in the E group. Participants in the R group had the highest HFD and SampEn values (HFD_Control_ < HFD_Episode_ < HFD_Remission_, *p* < 0.01 for each comparison; SampEn_Control_ < SampEn_Episode_ < SampEn_Remission_, *p* < 0.01 for each comparison).

**Figure 3:**
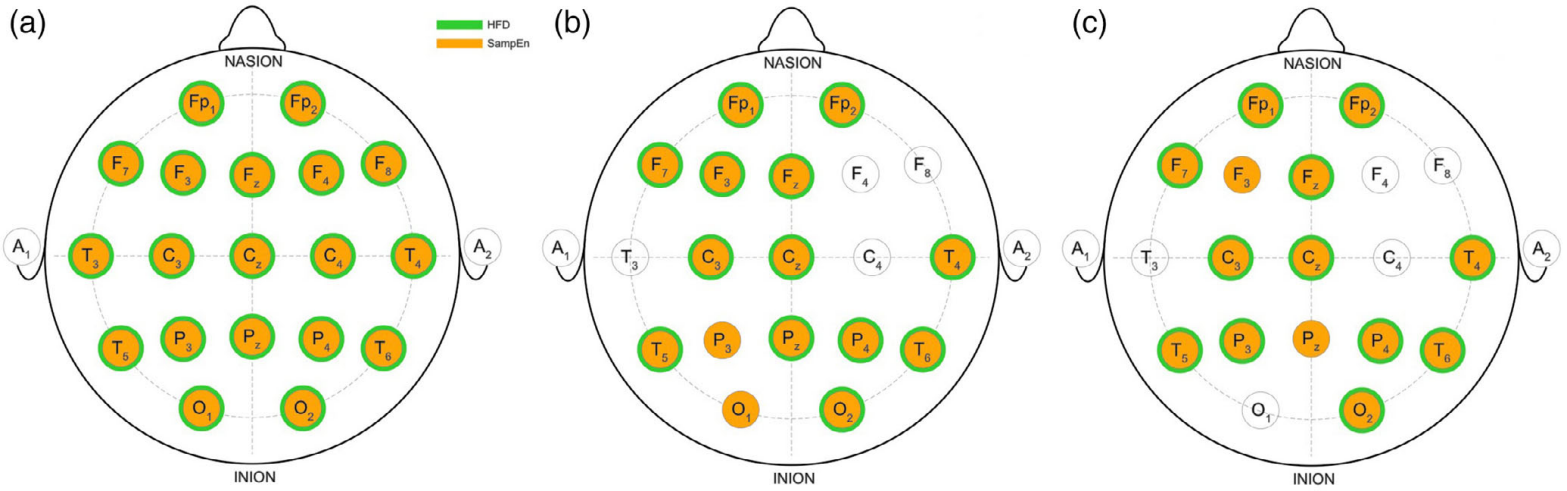
The spatial representation of significant differences in HFD and SampEn. In orange, electrodes from which SampEn values showed a significant difference in comparison to controls, and in green electrodes where HFD had a significant difference. The left panel (A) shows the difference between the whole group of patients (without splitting into E and R group) compared to controls. The middle panel (B) shows the comparison of all three groups (C vs. E vs. R); electrodes with statistically significant difference between each of three groups are marked (HFD/SampEn(C) < HFD/SampEn (E) < HFD/SampEn (R)). A significant difference was found on Fp1, Fp2, F7, F3, Fz, Cz, C3, P3, P4, Pz, T4, T5, T6, O1, and O2 electrodes for SampEn, and on Fp1, Fp2, F7, F3, Fz, Cz, C3, P4, Pz, T4, T5, T6, and O2 electrodes for HFD. Right panel (C) shows the comparison of E and R groups. SampEn values were significantly different on Fp1, Fp2, F7, F3, Fz, C3, Cz, P3, P4, Pz, T4, T5, T6, and O2 electrodes. HFD values were significantly different on Fp1, Fp2, F7, Fz, C3, Cz, P3, P4, T4, T5, T6, and O2 electrodes. *p* < 0.05. HFD, Higuchi's fractal dimension

The final level of analysis was to determine the possible effect of a group on HFD and SampEn values for each electrode. Figure 3a shows that for both measures there is a significant difference between the patient and control group (both E and R together). ANOVA showed a significant effect of Group (C, E, R) on HFD values for each electrode (*p* < 0.01).

However, post hoc test showed that significant difference between each group was found for electrodes Fp1, Fp2, F3, F7, Fz, C3, Cz, P4, Pz, T4, T5, T6, and O2 (*p* < 0.05) (Figure 3b). Post hoc Bonferroni correction showed that for each electrode HFD_Control_ < HFD_Episode_ < HFD_Remission_, *p* < 0.05 for each comparison. A similar result was found for SampEn values. ANOVA showed a significant effect of group (C, E, R) on SampEn values for each electrode (*p* < 0.01). Post hoc showed that this difference is driven by significantly lower SampEn values in C group when compared to E and R groups. However, the post hoc test showed that significant difference between each group was found for electrodes Fp1, Fp2, F3, F7, Fz, C3, Cz, P3, P4, Pz, T4, T5, T6, O1, and O2 (*p* < 0.05) and that for each electrode SampEn_Control_ < SampEn_Episode_ < SampEn_Remission_, *p* < 0.05 for each comparison.

SampEn showed to discriminate R and E groups on more electrodes compared to HFD. SampEn differed between R and E groups on Fp1, Fp2, F3, F7, Fz, C3, Cz, P3, P4, Pz, T4, T5, T6, and O2 electrodes, while HFD was different on Fp1, Fp2, F7, Fz, C3, Cz, P3, P4, T4, T5, T6, and O2 electrodes (*p* < 0.05 for each significant difference) (Figure 3c). For each comparison, patients in an acute episode had lower HFD and SampEn values compared to patients in a remission phase.

**Figure 4:**
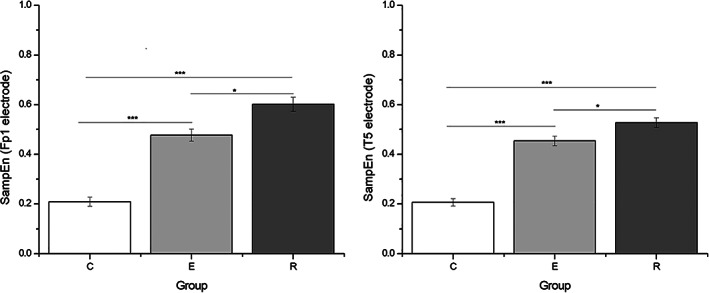
Results of (ANOVA) comparison of calculated sample entropy for certain electrodes which are particularly well discriminative: left, the values of SampEn for position Fp1 (fronto‐parietal), and right, the values of calculated SampEn for EEG recorded from position T5 (temporal). The significant difference between participants with depression and healthy control is particularly pronounced, and the difference between exacerbation (acute episode) and remission is also found

Figure 4 shows a representative result of a SampEn trend for Fp1 and T5 region in all three groups (C < E < R).

### 3.3 Principal component analysis (PCA)

**Figure 5:**
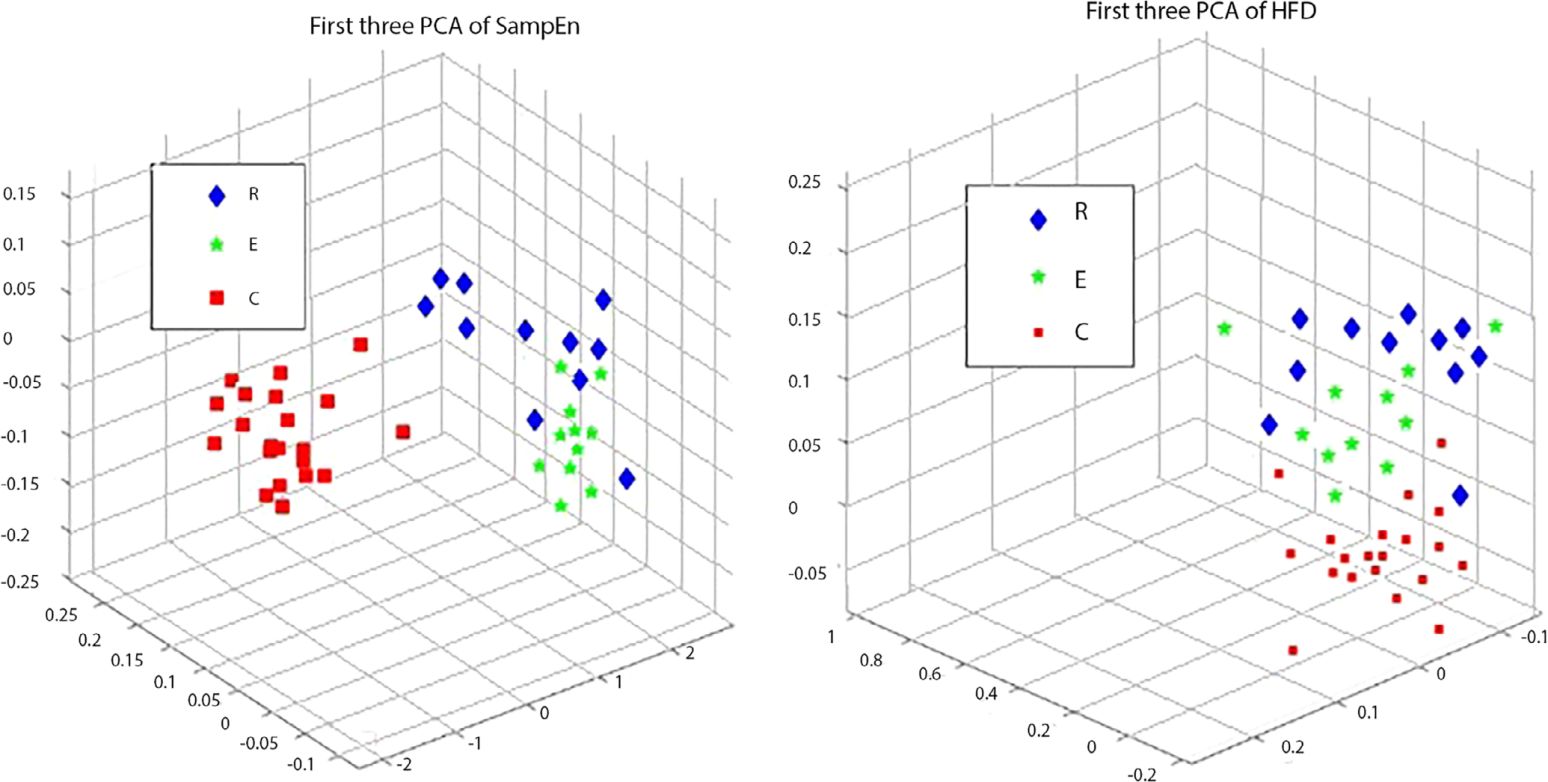
Principal component analysis was used to show the separability of the data. We used only first three principal components. Blue diamonds are symbols for those who are diagnosed with depression and are in remission; green stars are depressed patients who are in exacerbation and red squares are representing control group. Panel left represents how separable are the values of SampEn calculated from EEG of subjects from these three groups; panel on the right represents the values of calculated. HFD, Higuchi's fractal dimension

With only the first three PCs we want here to illustrate that those calculated values are separable. Again, SampEn gave more clear separation of the data when compared to HFD (in Figure 5, SampEn results are on the left and HFD on the right picture). In this study, we did not intend to deal with further classification, although a high accuracy could be obtained for several machine learning algorithms.

## 4 DISCUSSION

Our results show that both HFD and SampEn nonlinear measures of resting state EEG signal discriminate between healthy controls and depressed patients. These differences are widely distributed and include frontal, midline (vertex), and temporal–parietal–occipital regions. Furthermore, the complexity differs significantly between episode and remission, being higher in remission than in the episode phase of the disease.

Although this last finding is counterintuitive (it would be expected that the remission state is closer to the healthy one in every respect), it might be in line with the observation of Willner, Scheel‐Krüger, and Belzung (2013): “It is evident that antidepressants do not normalize brain activity: mood and behavior are restored to normal, but the antidepressant‐treated brain is in a different state from the nondepressed brain.” In line with this is also a conclusion of a recent review on complexity research in depression, that MDD dynamics might “appear more complex but is actually more random,” which has a particularly supported meaning grounded in the theory of information (de la Torre‐Luque & Bornas, 2017). Now, it is even in a linguistic sense complicated to explain what “complexity” means (Vargas, Cuesta‐Frau, Ruiz‐Esteban, Cirugeda, & Varela, 2015). What is the difference between healthy complexity (the level of chaos inevitable to adapt and survive) in contrast to pathological complexity (which is too much or too low)? In one side of spectrum are very simple (usually man‐made) electrical signals like sine wave which are completely predictable; on the other side is totally random white noise. Everything living is producing electrical signals which are somewhere in between those two opposed values. We still have to learn what is the level of complexity characteristic for many signals representing healthy processes, and those which are characteristic for so called disorder (even here we can see the trace of formerly dominant approach in physiology, teaching that everything is in “order,” linear, regular, and in equilibrium). There are scarce findings from other area of research supporting the understanding of those results. Lebiecka et al. (2018) examined (by applying HFD analysis) the levels of complexity in EEG in persons who were treated with rTMS due to their treatment‐resistant depression (TRD). In those who showed to be responders to the therapy increased physiological complexity fell significantly after the treatment, illustrating that this particular modality of stimulation is elevating their highly excitable (or perhaps, more random) dynamical system. When comparing the results of varying methods probing the complexity in depression, there are plenty of very different (in a mathematical sense) algorithms, but the common denominator is that majority of them are pointing to an increased physiological complexity as a characteristic of this functional disorder. In that sense, the two nonlinear measures we chose to apply showed to be methodologically a good pair capable of application in real time, because they are computationally fast.

According to the research on the first‐episode depression, such brain is initially different from the nondepressed one and the differences are structural (Ramezani et al., 2015), as well as functional (Bluhm et al., 2009; Zhang et al., 2011). In terms of nonlinear dynamic systems, initial conditions of the depressed brain system are different from the nondepressed. Even if the dynamics of information processing were the same, these different initial conditions would direct the system to steady states distinctive from normal. However, the dynamics is probably also different due to compensatory mechanisms. For example, functional neuroimaging revealed reduced integrity of the uncinate fasciculus and enhanced functional connectivity of anterior cingulate cortex and medial temporal lobe in MDD (de Kwaasteniet et al., 2013). The higher severity of depression, the more pronounced this negative structure–function relation. The authors suggest that the increased functional connectivity is a compensatory mechanism for decreased uncinate fasciculus integrity. Willner et al. (2013) came to a similar conclusion that decreased hippocampal functioning in depression causes an increase in the activity of the ventral “affective” system. It is then easy to suppose that the enrichment of fronto‐limbic connectivity and reorganization of circuits is accompanied by increment in complexity.

The EEG hallmark of depression is the presence of stable hemispheric asymmetry in the alpha spectral band, although the differences in other spectral bands were also demonstrated (Gold, Fachner, Erkilla, & Erkkila, 2012). Interestingly, van der Vinne, Vollebregt, van Putten, and Arns (2017) showed that frontal alpha asymmetry cannot be used as biomarker. Other researchers relying solely in spectral analysis also confirmed the existence of specific spectral structures in depression contrary to controls (Fingelkruis et al., 2014; Fingelkurts et al., 2006; Fingelkurts & Fingelkurts, 2015). Our spectral analysis shows that the power was decreased in alpha and high‐alpha bands in majority of the cortical regions, but increased in beta bands in posterior regions in patients. This may point towards the presence of hyperactivity in posterior regions (alpha desynchronization) of the right hemisphere, which is known to process the negative emotional content (Coan & Allen, 2004).

How we can compare our results to other researchers' findings? When it is so simple, why everyone is not using it already? One important aspect pertains to methodological differences between the studies, related to signal acquisition (number of EEG electrodes used, sampling frequency, pre‐processing of raw signal, that is, decomposition on bands and filtering), as well as experimental design (probing the emotional content, using different stimuli, performing cognitive task, etc.). The eyes‐closed condition, unlike the eyes‐open condition, allows measurement of the resting state arousal without the influence of cortical processing of the visual input in other bands on the complexity of brain dynamics. Also, it should be noted that we did not divide the spectrum of the signal to standard bands, but observed the changes in broadband. This is important as it has been shown that signal decomposition like Fourier, Wavelet, or cosine transformation can impact the result of a subsequent nonlinear analysis yielding erroneous results (Klonowski, 2007; Rabinovich, Varona, Selverston, & Abarbanel, 2006). Other reasons may relate to inherent differences between nonlinear algorithms that are based on different theoretical frameworks (Goldberger, Peng, & Lipsitz, 2002; Pincus & Goldberger, 1994). Our results are in line with studies that also used HFD (Ahmadlou et al., 2012; Bachmann et al., 2013; Acharya et al., 2015). The difference in complexity values between depressed and healthy subjects in our study was much larger than those reported in Bachmann et al., 2013. Another possible source of difference is choosing different values for k in Higuchi's FD algorithm; Bachmann et al. (2013) used 50 for their *k* value in that particular research (and ours was eight) making the comparison difficult.

The results of Fractal and SampEn maps are in line with previous electrophysiological studies demonstrating the presence of stable frontal asymmetry (Allen, Urry, Hitt, & Coan, 2004; Davidson, 2004) in MDD. However, in this study the signal was not divided to standard bands, hindering conclusion that current findings are directly related to the alpha band asymmetry. The results point to elevated complexity in frontal, central and right parieto‐temporal regions. This is also in line with earlier EEG studies (Haase et al., 2014), which reported similar topographical changes in distribution.

It should be noted that we used HFD and SampEn, two nonlinear measures able to detect differential aspects of the signal under analysis. While HFD examines the complexity in the time domain, SampEn can characterize the irregularity of a signal or its predictability (indicated indirectly as complexity changes in physical sense). They both showed higher complexity in patients with depression when compared to healthy control subjects. The difference was more pronounced when examined by SampEn suggesting increased variability, or “irregularity” or unpredictability or even randomness of the signal. Further study of this paritcular difference is needed on a larger sample. But the presented result is showing that this possible differentiation which can be utilized in real‐time situations holds a promisse for future clinical application.

## 5 CONCLUSIONS

The idea of EEG‐based classification of depression is not entirely novel. Our study confirmed that it is possible to quantify the difference between depressed patients and controls by employing two complexity measures—HFD and SampEn, on resting state EEG. Furthermore, it showed, for the first time, that both measures could detect a statistically significant difference between depressed patients who were in episode and remission. Whether these and other nonlinear measures may be used as potential clinical markers of disease stage or of the effectiveness of various treatments in MDD remains to be confirmed on larger groups of patients. Finally, we are well aware of the need for further thorough probing of the research methodology. It is always possible, when dealing with the human EEG in psychiatric disorders, that some uncontrolled variable could make false positive/negative results. Comparing different methods together with more rigorous sample selection criteria, in the EEG signal analysis would shed more light on this important problem.

## DECLARATION OF INTEREST STATEMENT

The authors declare no conflict of interest.
